# Evaluation of the Magicplex™ Sepsis Real-Time Test for the Rapid Diagnosis of Bloodstream Infections in Adults

**DOI:** 10.3389/fcimb.2019.00056

**Published:** 2019-03-12

**Authors:** Yuliya Zboromyrska, Catia Cillóniz, Nazaret Cobos-Trigueros, Manel Almela, Juan Carlos Hurtado, Andrea Vergara, Caterina Mata, Alex Soriano, Josep Mensa, Francesc Marco, Jordi Vila

**Affiliations:** ^1^The Consortium of the Intercomarcal Laboratory of the Alt Penedès, Department of Microbiology, Vilafranca del Penedès, Spain; ^2^Department of Pneumology, Hospital Clinic of Barcelona, Institut d'Investigacions Biomèdiques August Pi i Sunyer (IDIBAPS), Networked Biomedical Research Center for Respiratory Diseases (CIBERES), University of Barcelona, Barcelona, Spain; ^3^Department of Infectious Diseases, Hospital Clinic, University of Barcelona, Barcelona, Spain; ^4^Department of Microbiology, ISGlobal, Barcelona Centre for International Health Research, Hospital Clinic, University of Barcelona, Barcelona, Spain; ^5^Sample Preparation Team, Centre Nacional d'Anàlisi Genòmica, Parc Científic de Barcelona – Torre I, Barcelona, Spain

**Keywords:** bloodstream infection, PCR-based assay, blood culture, Magicplex™ Sepsis test, infection, sepsis

## Abstract

Sepsis is a serious health condition worldwide, affecting more than 30 million people globally each year. Blood culture (BC) is generally used to diagnose sepsis because of the low quantity of microbes occurring in the blood during such infections. However, ~50% of bloodstream infections (BSI) give negative BC, this figure being higher for sepsis, which delays the start of appropriate antimicrobial therapy. This prospective study evaluated a multiplex real-time polymerase chain reaction, the Magicplex^TM^ Sepsis test (MP), for the detection of pathogens from whole blood, comparing it to routine BC. We analyzed 809 blood samples from 636 adult patients, with 132/809 (16.3%) of the samples positive for one or more relevant microorganism according to BC and/or MP. The sensitivity and specificity of MP were 29 and 95%, respectively, while the level of agreement between BC and MP was 87%. The rate of contaminated samples was higher for BC (10%) than MP (4.8%) (*P* < 0.001). Patients with only MP-positive samples were more likely to be on antimicrobial treatment (47%) than those with only BC-positive samples (18%) (*P* = 0.002). In summary, the MP test could be useful in some clinical setting, such as among patients on antibiotic therapy. Nevertheless, a low sensitivity demonstrated impairs its use as a part of a routine diagnostic algorithm.

## Introduction

The World Health Organization (WHO) in 2017 adopted a resolution on sepsis: “improving the prevention, diagnosis and clinical management of sepsis” (World Health Organization, 2018). Blood culture (BC) is generally used to diagnose sepsis because of the low quantity of microbes occurring in the blood during such infections. However, ~50% of bloodstream infections (BSI) give negative BC, this figure being higher for sepsis, which delays the start of appropriate antimicrobial therapy and consequently results in worse outcomes and higher mortality rates (Kumar et al., 2006; Ferrer et al., 2014). Prompt microbiological diagnosis enables a more appropriate antimicrobial treatment than the empirical combination of broad-spectrum antibiotics that have negative effects such as an increased prevalence of resistant pathogens (Candel et al., 2018). The microbial diagnosis of sepsis by BC has two main advantages: first, it allows the growth of very small numbers of the microorganism, which is important since the concentration of bacteria in the blood of septic adult patients is usually low (< 10 CFU/mL) (Yagupsky and Nolte, 1990); second, this technique allows the isolation of pathogens and, hence, antimicrobial susceptibility testing can be performed. However, BC also has limitations that do not make it an ideal gold standard test, including the long time required for growth detection, the frequent false negative results in patients receiving antimicrobial therapy, and the inability to detect fastidious microorganisms (Sinha et al., 2018) which can delay the initiation of an adequate antimicrobial therapy and consequently results in worse outcomes and higher mortality rates (Kumar et al., 2006; Ferrer et al., 2014). Molecular tests offer important advantages over BC that could improve the diagnosis of BSI, such as the lower amount of time taken to obtain results by working directly from blood. In addition, the low detection limits of molecular assays might make them more sensitive than BC, enabling the detection of fastidious, non-viable or non-culturable microorganisms, even from patients on antibiotic treatment (Fenollar and Raoult, 2007; Leitner et al., 2013; Sinha et al., 2018).

Another advantage of molecular assays is the ability to detect some specific resistance markers, which can provide important information for better treatment. Moreover, the rapid identification of the microorganism can be used to infer antimicrobial susceptibility according to local epidemiology.

The Magicplex^TM^ Sepsis test (MP) (Seegene, Seoul, South Korea) is a multiplex real-time polymerase chain reaction (PCR) that detects more than 90 microorganisms at the genus level (73 Gram-positive bacteria, 12 Gram-negative bacteria and 6 fungi), 27 at the species level and 3 drug-resistant genes (*mecA, vanA*, and *vanB)* within 6 h. In this study, we evaluated the ability of the MP test to rapidly detect pathogens causing BSI in adult patients from whole blood compared to conventional BC.

## Methods

### Setting, Data, and Sample Collection

The study was approved by the Ethics Committee of the Hospital Clinic of Barcelona (study no. 2011/6613). Written informed consent was waived because of the non-interventional study design.

Paired BC and 1 ml samples of whole blood in an EDTA tube were obtained from adult patients (≥18 years old) from the Hospital Clinic of Barcelona, a 700-bed university hospital in Barcelona, Spain, from May to September 2011.

Samples were obtained from patients with suspected BSI and who met the criteria for BC collection. Samples were processed in parallel by MP and BC. Blood samples for MP and BC were obtained simultaneously from the same catheter or venipuncture. For each case, additional clinical data about ongoing antibiotic treatment, the suspected source of infection, as well as the results of other microbiological tests were collected.

### Routine Microbiological Techniques

BC was incubated in a Bactec 9240® (Becton Dickinson, MD, USA) for a maximum of 5 days. The following bottles were used: the resin-containing BACTEC Plus Aerobic/F and BACTEC Plus Anaerobic/F, and the non-resin-containing BACTEC Standard/10 Aerobic/F and BACTEC Lytic/10 Anaerobic/F. For positive samples, Gram staining and culturing on solid media were performed. Microorganisms were identified using matrix-assisted laser desorption/ionization time-of-flight mass spectrometry (MALDI-TOF MS) (Bruker Daltonics, Bremen, Germany). Routine susceptibility testing included the Phoenix™ system (Becton Dickinson, MD, USA), Etest (AB bioMérieux), microdilution (Sensititre, Trek Diagnostic Systems, Inc., Westlake, Ohio, USA) and disc diffusion tests, depending on the pathogen isolated. Determination of the isolates as contaminants or pathogens was achieved by combining the clinical setting, pathogenicity of the isolated microorganisms and the number of positive BC bottles in the case of potential skin contaminants, such as coagulase-negative staphylococci (CoNS). CoNS were considered pathogens if the same species was detected in both sets of BC showing the same antimicrobial susceptibility pattern. Samples with positive BC for pathogens not included in the MP panel were excluded from the analysis.

### The Magicplex™ Sepsis Test (MP)

**Figure 1:**
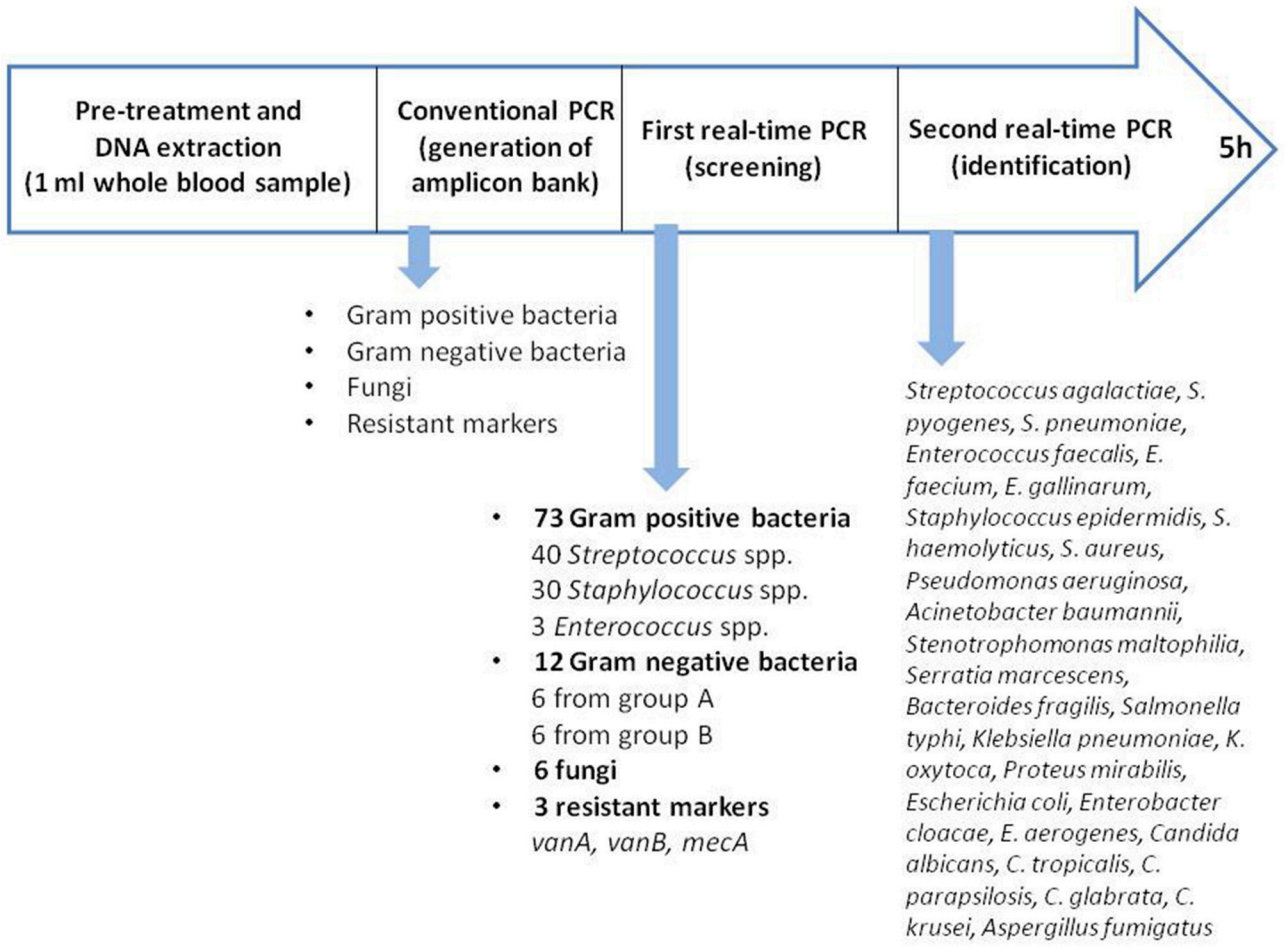
Process of Magicplex™ sepsis.

Blood samples were initially pre-treated with the Seegene Blood Pathogen Kit™, according to the manufacturer's instructions. This pre-treatment included the following steps: lysis of human cells with CM buffer, degradation of released DNA with MolDNase B, concentration by centrifugation of microorganisms and, finally, lysis of cells and release of nucleic acid. Bacterial DNA was automatically extracted and purified with the SeePrep12™ extractor (Seegene). Amplification, screening and identification were performed following the manufacturer's instructions. Each sample was first processed by two conventional PCRs performed in two separate tubes: one for the detection of Gram-positive bacteria and resistance genes, and the other for Gram-negative bacteria (GNB) and fungi (Figure 1).

For this step the thermal cycler System 9700, Applied Biosystems was used. The first real-time PCR for screening was performed in three separate tubes, allowing the identification of pathogens at the genus or group level: *Streptococcus* spp., *Staphylococcus* spp., *Enterococcus* spp., GNB group A or B, fungi and 3 resistance genes (*vanA, vanB*, and *mecA*). For positive results, the second real-time PCR was carried out, allowing the identification of 27 pathogens at the species level, including 3 *Streptococcus* spp. (*Streptococcus pneumoniae, Streptococcus agalactiae* and *Streptococcus pyogenes*), 3 *Enterococcus* spp. (*Enterococcus faecalis, Enterococcus faecium* and *Enterococcus gallinarum*), 3 *Staphylococcus* spp. (*Staphylococcus epidermidis, Staphylococcus haemolyticus* and *Staphylococcus aureus*), 6 GNB from group A (*Pseudomonas aeruginosa, Acinetobacter baumannii, Stenotrophomonas maltophilia, Serratia marcescens, Bacteroides fragilis*, and *Salmonella typhi*), 6 GNB from group B (*Klebsiella pneumoniae, Klebsiella oxytoca, Proteus mirabilis, Escherichia coli, Enterobacter cloacae*, and *Enterobacter aerogenes*), and 6 fungi (*Candida albicans, Candida parapsilosis, Candida glabrata, Candida tropicalis, Candida krusei*, and *Aspergillus fumigatus*). The real-time PCR were performed using the Cepheid SmartCycler.

The Seegene Viewer software was used to interpret the results, which were available within 6 h. All the samples were stored at 2–8°C and processed within 24 h after collection.

MP samples positive for CoNS were considered to be true positives if the same microorganism was detected in two sets of BC. Samples that were positive according to MP in the first real-time PCR screening and identified only at the group level (e.g., GNB group A), but negative in the second real time-PCR were considered negative according to MP. To avoid confusing calculation in sensitivity and specificity we consider samples with only contaminants detected by MP and/or BC as negative. Internal control is included in each PCR step. Microorganisms identified by MP only at the genus level, such as *Staphylococcus* spp. and *Streptococcus* spp., were considered to be contaminants if the BC was negative or positive for staphylococci or streptococci that could be identified by MP at the species level.

### Statistical Analysis

Categorical variables were compared using the chi-square test. To calculate the yield of the test, we analyzed sensitivity and specificity, and predictive values. All statistical comparisons were two-sided hypothesis tests, and the significance level was set at 0.05. All confidence intervals (CIs) were two-sided at 95% confidence level. All analyses were performed with IBM SPSS Statistics 22.0 (Armonk, New York).

## Results

A total of 809 samples from 636 adult patients were collected and analyzed. Among the samples, 435 out of 809 (54%) were from the emergency wards, 197 (24%) from intensive care units (ICUs) and surgery, 159 (20%) from oncology and hematology wards, and 18 (2%) from other medical wards. The average age of patients was 56.7 ± 5.6; 43% were females.

A total of 140 pathogens were detected among 132 positive samples (Table 1). *E. coli* was the most frequently detected pathogen (47/140, 33.6%), followed by CoNS (16/140, 11.4%), *Candida* spp. (14/140, 10%), and *S. aureus* (13/140, 9.3%).

**Table 1:** Comparison of microbiological results between BC and MP.

**Pathogens detected**	**Positive only BC**	**Positive only MP**	**Positive both methods**	**Total, *n* (%)**	**Prevalence among 809 samples, %**
*Escherichia coli*	27	15	5	47 (34)	5.8
CoNS	7	0	9	16 (11)	2
*Candida* spp.	9	2	3	14 (10)	1.7
*Staphylococcus aureus*	2	7	4	13 (9)	1.6
*Enterococcus faecalis*	5	3	3	11 (8)	1.4
*Pseudomonas aeruginosa*	6	2	1	9 (6)	1.1
*Klebsiella pneumoniae*	6	2	1	9 (6)	1.1
*Streptococcus* spp.	3^a^	2^b^	1^c^	6 (4)	0.7
*Enterobacter cloacae*	3	1	0	4 (3)	0.5
*Enterococcus faecium*	2	0	1	3 (2)	0.4
*Acinetobacter* spp.	1	2	0	3 (2)	0.4
*Serratia marcescens*	1	1	0	2 (2)	0.2
*Enterococcus gallinarum*	0	1	0	1 (1)	0.1
*Klebsiella oxytoca*	0	1	0	1 (1)	0.1
*Stenotrophomonas maltophilia*	0	1	0	1 (1)	0.1
Total, *n* (%)	72 (51)	40 (29)	28 (20)	140 (100)	

*BC, blood culture; MP, MagicPlex assay; CoNs, coagulase-negative staphylococci*.

a *Streptococci of viridians group (S. anginosus and S. gordonii) and one S. agalactiae*.

b *One S. agalactiae and one S. pneumoniae*.

c *S. constellatus*.

BC identified 3 mixed infections: *E. cloacae* plus *K. pneumonia*e, *C. glabrata* plus *S. haemolyticus*, and *P. aeruginosa* plus *S. epidermidis*. MP detected 4 mixed infections: *S. agalactiae* plus *S. aureus, E. coli* plus *S. epidermidis, P. aeruginosa* plus *S. marcescens*, and *E. coli* plus *E. gallinarum*. One mixed infection (*E. faecium* plus *S. haemolyticus*) was identified by both methods.

Three *mecA* genes were detected among the 9 pathogenic CoNS identified by the two methods. Routine susceptibility testing detected 7 methicillin-resistant CoNS and 2 methicillin-susceptible strains. Only one *vanB* resistance marker was detected in a sample positive for *E. faecalis* only according to MP and not BC. Therefore, this result remained unconfirmed. No additional BC or other microbiological samples positive for *E. faecalis* were obtained from this patient.

As shown in Table 2, samples with microbial contaminants were more frequently observed with BC than MP: 81/809 (10%) vs. 39/809 (4.8%) (*P* <0.001), respectively.

**Table 2:** Concordance of the results obtained by BC and MP.

	**Number of samples**			
	**BC positive**	**BC negative**	**BC-contaminated**	**Total**
MP-positive	28	31	5	64
MP-negative	62	574	70	706
MP-contaminated	6	27	6	39
Total	96	632	81	809

*BC, blood culture; MP, MagicPlex assay*.

Blood samples in which only microbial contaminants were detected were considered as negative for further analysis. As a result, 132/809 (16.3%) of the samples were considered positive for one or more relevant microorganism by BC and/or MP. The level of agreement between BC and MP was 87.1% (705/809). Regarding the 28 samples positive according to both methods, the same pathogen was identified in 27 of the cases, with the remaining one sample showing inconsistent results: *E. coli* detected by MP and *E. faecium* by BC. The rate of positive results was higher for BC (96/809, 11.9%) than MP (64/809, 7.9%). Considered BC the gold standard and considering negative the samples with only contaminants detected, the sensitivity, specificity, positive predictive value and negative predictive value of MP were 29.2% (95% CI, 20.6–39.5), 95% (95% CI, 93–96.4), 43.8% (95% CI, 31.6–56.7), and 90.9% (95% CI, 88.5–92.8), respectively.

Additional clinical information, including underlying disease and the antibiotic or antifungal therapy administered on the day of sampling, was recorded for the patients with 36 MP-positive and BC-negative or contaminated results (Table 3). When comparing the percentage of patients with undergoing antimicrobial treatment at the time of sample collection between three groups: only BC-positive (12/68, 17.6%), only MP-positive (17/36, 47.2%), and BC and MP-positive (8/28, 28.6%), there was significantly higher number of patients receiving antimicrobials in the group of only MP-positive (*P* = 0.002). Interestingly, 3 of the 17 patients with only an MP-positive sample had a BC positive for the same microorganism in the previous days, while in 5 patients, other microbiological samples were positive for the pathogen detected by MP.

**Table 3:** Additional information from 36 patients with MP-positive and BC-negative results.

**Setting**	**MP result**	**Clinical condition**	**Other microbiological tests positive for the same pathogen**	**Ongoing antibiotic/antifungal treatment^a^**
ICU and surgery (*n* = 16)	*Klebsiella oxytoca*	Self-limiting fever after removal of a Kehrs tube in a liver transplant patient	–	–
	*Escherichia coli*	Subarachnoid hemorrhage Control BC 3 days after catheter-related *Pseudomonas aeruginosa* bacteremia	–	Ciprofloxacin
	*Escherichia coli*	Necrotizing fasciitis	BC and wound swab culture positive for *E. coli* 3 days before sampling	Meropenem
	*Staphylococcus aureus*	Necrotizing fasciitis	BC positive for *S. aureus* 2 days before sampling	Cloxacillin
	*Streptococcus pneumoniae*	Acute myeloid leukemia with neutropenic fever without a clinical focus	–	Piperacillin-tazobactam, Vancomycin
	*Candida parapsilosis*	*K. pneumoniae* bacteremia secondary to mesenteric vein thrombosis in a cirrhotic patient Nosocomial pneumonia due to *S.aureus* and *Acinetobacter baumannii*	3 bronchial washing samples positive for *Candida* spp.	Fluconazole
	*Escherichia coli*	Septic shock due to *S. aureus* cellulitis in a cirrhotic patient. Ascites decompensation	Wound smear positive for *S. aureus*	Ceftazidime and Tigecycline
	*Escherichia coli*	Acute necrotizing pancreatitis with peripancreatic abscess	–	Meropenem
	*Staphylococcus aureus*	Superinfection of Pulmonary contusion in a polytrauma patient	Tracheal aspirate positive for *S. aureus* 2 days before sampling	Levofloxacin
	*Escherichia coli*	Colonic perforation post-nephrectomy	–	Piperacillin-tazobactam
	*Enterobacter cloacae*	Intestinal obstruction and abdominal sepsis secondary to inguinal hernia	–	Levofloxacin
	*Acinetobacter baumannii*	Post-operative fever (liver resection 48 h before sampling)	–	–
	*Acinetobacter baumannii*	Thoracic empyema in a cirrhotic patient	–	–
	*Staphylococcus aureus*	Hepatocellular carcinoma. Metabolic decompensation of diabetes mellitus	–	–
	*Escherichia coli*	Urinary tract infection (UTI)	–	Ciprofloxacin and Ceftriaxone
	*Candida parapsilosis*	Post-operative intraabdominal abscess	–	–
Emergency department (*n* = 15)	*Escherichia coli*	UTI	–	–
	*Staphylococcus aureus*	Stroke. Bronchoaspiration in a patient with prior colonization with *S. aureus*.	–	–
	*Streptococcus agalactiae, Staphylococcus aureus*	Cellulitis	–	–
	*Escherichia coli*	HIV patient with prosthetic aortic valve with progressive prurigo nodularis	–	Amoxicillin/clavulanic acid
	*Escherichia coli*	Fever during hemodialysis	Urine culture positive for *E. coli*	Amoxicillin/clavulanic acid
	*Escherichia coli*	Acute exacerbation of chronic obstructive pulmonary disease (COPD)	Sputum sample positive for *E. coli*	–
	*Enterococcus faecalis*	Traveler's diarrhea	–	–
	*Pseudomonas aeruginosa*	Acute gastroenteritis and community-acquired UTI	–	–
	*Staphylococcus aureus*	Pneumonia	–	–
	*Staphylococcus aureus*	UTI in a patient with permanent bladder catheter	–	Amoxicillin/clavulanic acid
	*Pseudomonas aeruginosa*	Acute exacerbation of Crohn's disease.	–	–
	*Klebsiella pneumoniae*	*Pneumocystis carinii* pneumonia in an AIDS patient	–	–
	*Stenotrophomonas maltophilia*	Community-acquired UTI	–	–
	*Escherichia coli*	Acute exacerbation of COPD	–	–
	*Klebsiella pneumoniae*	*Pneumocystis carinii* pneumonia in an AIDS patient. Bacteremia caused by *E. faecalis*	–	–
Oncology and hematology (*n* = 4)	*Enterococcus faecalis* (vanB positive)	Non-Hodgkin T-cell lymphoma. Neutropenic fever	–	Levofloxacin
	*Escherichia coli*	Acute myeloid leukemia. Neutropenic fever	–	–
	*Escherichia coli*	Stem cell transplanted patient due to acute myeloid leukemia. Neutropenic fever	–	–
	*Escherichia coli*	Fever in a patient with acute lymphoblastic leukemia	–	Meropenem
	*Enterococcus faecalis*	Catheter-related blood stream infection in a patient transplanted due to acute myeloid leukemia	BC positive for *E. faecalis* 6 days before sampling	Daptomycin

*ICU, intensive care unit; BC, blood culture; MP, MagicPlex assay*.

a *Only antimicrobial/antifungal therapy potentially effective against pathogen detected and initiated before sampling*.

## Discussion

Several molecular assays have been tested for the direct molecular identification of pathogens in blood samples (Lehmann et al., 2008; Wellinghausen et al., 2009; Bloos et al., 2012; Grif et al., 2012; Loonen et al., 2013; Nieman et al., 2016) However, few studies have focused on commercially available PCR-based tests other than the SeptiFast test for the detection of BSI (Kühn et al., 2011; Fitting et al., 2012). Recently, T2 magnetic resonance assay was evaluated to detect *Candida* spp. and some bacteria in whole blood (Mylonakis et al., 2015; Snyder et al., 2017; Peker et al., 2018). In the present study, we evaluated the Magicplex^TM^ Sepsis test (MP), comparing it with conventional BC. We observed that MP showed a sensitivity and specificity of 29.2 and 95%, respectively. Only a few studies have evaluated the sensitivity and specificity of MP (Carrara et al., 2013; Loonen et al., 2013; Ljungström et al., 2015; Denina et al., 2016; Ziegler et al., 2016). Carrara et al. (2013), assessing 267 patients from ICU, emergency and hematology wards, reported an overall sensitivity of 65% for MP compared with 41% for BC. However, the authors calculated the sensitivities and specificities using a reference standard in which clinical data and other cultures were added to the BC results to see if a positive MP result represented a true BSI or not. Loonen et al. (2013), investigating 125 patients from the emergency department, reported a sensitivity of 37% and a positive predictive value (PPV) of 30% for MP, the low PPV resulting from many of the samples being positive for CoNS according to MP. Ljungström et al. (2015), analyzing samples from 375 patients with suspected sepsis collected from emergency wards, excluded suspected contaminants such as CoNS. They reported a sensitivity of 64% and specificity of 96% for MP. However, if they had included all the MP and BC results, the sensitivity of MP would have been 38%, PPV 17%, and specificity 65%. Taking into account all these results, the MP test could be useful in some clinical setting, such as among patients with undergoing antimicrobial treatment and negative blood cultures. Nevertheless, a low sensitivity demonstrated limits its use as a part of routine diagnostic algorithm. The low sensitivity of MP is probably due to the low bacterial concentration in whole blood and the low sample volume of 1 ml used. There are several strategies intended to improve bacterial recovery, such as removing or significantly reducing the amount of human DNA present in the whole blood sample. Nevertheless, this step is already included in the MP and SepsiTest assays. Another possibility is to introduce an additional incubation step prior to extraction (Serra et al., 2012). Although this approach can increase microbial concentrations, it lengthens the whole process and compromises the main advantage of the molecular assays, which is the rapid procurement of results. Another strategy is to increase the initial volume of the blood sample (>1 ml) (Hansen et al., 2009), which has been reported to give promising results and greatly improve the detection rates of PCR-based assays (Loonen et al., 2013) or increase the amount of bacterial DNA, as shown by Trung et al. (2016).

We observed 36 samples that were positive according to MP and negative according to BC. This could have been due to the fact that almost half of these samples were obtained from patients on antibiotic treatment, which could explain the negative BC results. Unfortunately, the data about the start time and duration of antimicrobial treatment was not collected; therefore more detailed analysis of the treatment impact on BC and MP positivity could not be performed. The presence of cell-free DNA or non-viable microorganisms or even contaminating DNA could explain the other 19 discordant results (Opota et al., 2015).

Importantly, MP detected 5 out of the 14 *Candida* spp. and 11 out of the 13 *S. aureus* isolates in our study. Early detection of these two pathogens is essential due to the high mortality rates and risk of haematogenous complications associated with these microbes. In addition, some *Candida* requires a longer time for growth in BC than other pathogens, delaying the initiation of treatment by several days. *Candida* spp. was the third most frequently occurring pathogen detected in our study. As expected, most of the positive samples were obtained from ICU patients. We detected a small number of resistance genes. Although the three *mecA* genes detected by MP were confirmed by routine susceptibility testing, in four cases of CoNS methicillin-resistant by routine susceptibility testing *mecA* gene was not detected by MP. Furthermore, the only *vanB*-positive *E. faecalis* detected by MP was not isolated by BC. Therefore, the accuracy of MP in detecting resistance genes should be further evaluated and probably is necessary the second molecular assay to confirm discordant results due to low predictor value of some phenotypic testing for detection of methicillin resistance.

Despite the many advantages of molecular assays, these tests have several important limitations. First, these methods require specialized equipment and technical experience and are usually expensive. The MP test also has several manual steps that make it laborious and increase the risk of possible contamination. Moreover, its low sensitivity makes its implementation as a routine test difficult in clinical microbiology laboratories. Finally, the sensitivity and specificity of molecular assays vary according to the test, the extraction method, the algorithm used to evaluate the results, the comparative method and the study population. Data published to date support the use of PCR-based tests for specific groups of patients, such as critically ill patients in ICU, those with suspected candidaemia or patients receiving broad-spectrum antibiotics (von Lilienfeld-Toal et al., 2009; Bravo et al., 2011). There is no consensus about the interpretation of BC-negative and PCR-positive results (low pathogen concentrations, non-viable bacteria and DNAemia, etc.). Further studies are needed to evaluate the role of new molecular assays in the routine microbiological diagnosis of BSI. The main strengths of our study are: the higher sample number comparing with previous reports; patients included were not only from emergency departments but also from ICU and oncology/hematology; clinical conditions and antimicrobial treatment of patients with positive results detected only by PCR was investigated.

The main limitation of this study is that discordant only MP positive results were considered as false positive due to the use of BC as comparator. BC lacks sensitivity and the second molecular assay should be used to evaluate discordant results.

In conclusion, sepsis is a time-dependent disease that requires early diagnosis and prompt appropriate treatment to improve prognosis. The MP assay provides results within 6 h, thereby significantly reducing the amount of time for diagnosis and seems to be especially useful in patients on antimicrobial treatment. Nevertheless, the assay has to be optimized, mainly to improve the sensitivity and also including other significant microorganisms within the panel. Greater automation is necessary to facilitate introduction of the assay into routine laboratory workflow and to reduce turnaround time. Furthermore, the microbial detection limits of the molecular assay need to be improved, probably by new extraction protocols and greater sample volumes to improve the positive and negative predictive power so that it can be a useful tool in clinical practice, especially regarding its impact on antibiotic use.

## Data Availability

The raw data supporting the conclusions of this manuscript will be made available by the authors, without undue reservation, to any qualified researcher. Requests to access the datasets should be directed to Catia Cillóniz.

## Author Contributions

CC, YZ, and JV had full access to all data in the study, and take full responsibility for its integrity, accuracy, and analysis. YZ, CC, NC-T, JH, MA, AV, CM, AS, JM, FM, and JV study concept and design, and acquisition, analysis, or interpretation of data, and critical revision of the manuscript for important intellectual content, and administrative, technical, or material support. CC, YZ, and JV drafting of the manuscript and study supervision, and statistical analysis.

### Conflict of Interest Statement

The authors declare that the research was conducted in the absence of any commercial or financial relationships that could be construed as a potential conflict of interest.
